# Study Protocol for the Development of a European eHealth Platform to Improve Quality of Life in Individuals With Huntington's Disease and Their Partners (HD-eHelp Study): A User-Centered Design Approach

**DOI:** 10.3389/fneur.2021.719460

**Published:** 2021-09-13

**Authors:** Pearl J. C. van Lonkhuizen, Niko J. H. Vegt, Eline Meijer, Erik van Duijn, Susanne T. de Bot, Jiří Klempíř, Wiebke Frank, G. Bernhard Landwehrmeyer, Alzbeta Mühlbäck, Jennifer Hoblyn, Ferdinando Squitieri, Peter Foley, Niels H. Chavannes, Anne-Wil Heemskerk

**Affiliations:** ^1^Department of Public Health and Primary Care, Leiden University Medical Center, Leiden, Netherlands; ^2^National eHealth Living Lab, Leiden University Medical Center, Leiden, Netherlands; ^3^Huntington Center Topaz Overduin, Katwijk, Netherlands; ^4^Department of Psychiatry, Leiden University Medical Center, Leiden, Netherlands; ^5^Department of Neurology, Leiden University Medical Center, Leiden, Netherlands; ^6^Department of Neurology and Center of Clinical Neuroscience, 1st Faculty of Medicine, Charles University and General University Hospital in Prague, Prague, Czechia; ^7^Department of Neurology, University Hospital Ulm, Ulm, Germany; ^8^Bloomfield Hospital, Trinity College Dublin, Dublin, Ireland; ^9^Huntington and Rare Diseases Unit, Fondazione Istituto di Ricovero e Cura a Carattere Scientifico (IRCCS) Casa Sollievo della Sofferenza Research Hospital, San Giovanni Rotondo, Italy; ^10^Department of Clinical Neurosciences, Anne Rowling Regenerative Neurology Clinic, University of Edinburgh, Edinburgh, United Kingdom

**Keywords:** Huntington disease, neurodegenerative diseases, telemedicine, eHealth, user-centered design, quality of life, study protocol, tele-neurology

## Abstract

**Background:** Huntington's disease (HD) is an autosomal dominant neurodegenerative disease that affects the quality of life (QoL) of HD gene expansion carriers (HDGECs) and their partners. Although HD expertise centers have been emerging across Europe, there are still some important barriers to care provision for those affected by this rare disease, including transportation costs, geographic distance of centers, and availability/accessibility of these services in general. eHealth seems promising in overcoming these barriers, yet research on eHealth in HD is limited and fails to use telehealth services specifically designed to fit the perspectives and expectations of HDGECs and their families. In the European HD-eHelp study, we aim to capture the needs and wishes of HDGECs, partners of HDGECs, and health care providers (HCPs) in order to develop a multinational eHealth platform targeting QoL of both HDGECs and partners at home.

**Methods:** We will employ a participatory user-centered design (UCD) approach, which focusses on an in-depth understanding of the end-users' needs and their contexts. Premanifest and manifest adult HDGECs (*n* = 76), partners of HDGECs (*n* = 76), and HCPs (*n* = 76) will be involved as end-users in all three phases of the research and design process: (1) Exploration and mapping of the end-users' needs, experiences and wishes; (2) Development of concepts in collaboration with end-users to ensure desirability; (3) Detailing of final prototype with quick review rounds by end-users to create a positive user-experience. This study will be conducted in the Netherlands, Germany, Czech Republic, Italy, and Ireland to develop and test a multilingual platform that is suitable in different healthcare systems and cultural contexts.

**Discussion:** Following the principles of UCD, an innovative European eHealth platform will be developed that addresses the needs and wishes of HDGECs, partners and HCPs. This allows for high-quality, tailored care to be moved partially into the participants' home, thereby circumventing some barriers in current HD care provision. By actively involving end-users in all design decisions, the platform will be tailored to the end-users' unique requirements, which can be considered pivotal in eHealth services for a disease as complex and rare as HD.

## General Information

### Protocol Title

Development of an eHealth care model to improve quality of life in Huntington's Disease: a user-centered design study (HD-eHelp).

### Research Sites and Investigators

Research sites from the Netherlands, Germany, Czech Republic, Italy, and Ireland will be involved in this study. Table 1 provides an overview of the participating research sites and investigators within the European eHealth Care Model for Rare Neurodegenerative Diseases (HEALTHE-RND) consortium.

**Table 1:** Research sites and investigators involved in the study.

**Investigators**	**Role**	**Research site**
**The Netherlands**		
P. J. C. van Lonkhuizen, MSc	Coordinating investigator	LUMC^*^, Topaz, NeLL
N. J. H. Vegt, PhD	Co-investigator	LUMC^*^, NeLL
E. Meijer, PhD	Co-investigator	LUMC^*^, NeLL
A. Heemskerk, PhD	Co-investigator	LUMC^*^, Topaz
E. van Duijn, MD, PhD	Co-investigator	LUMC^$^, Topaz
S. T. de Bot, MD, PhD	Co-investigator	LUMC^∇^
N. H. Chavannes, MD, PhD	Principal investigator	LUMC^*^, NeLL
**Germany**		
G. B. Landwehrmeyer, MD, PhD	Principal investigator	University Hospital Ulm
A. Mühlbäck, MD	Coordinating investigator	University Hospital Ulm
W. Frank, MSc	Coordinating investigator	University Hospital Ulm
R. Hoffmann, MD	Co-investigator	University Hospital Ulm
**Czech Republic**		
J. Klempíř, MD, PhD	Principal investigator	Charles University Prague
K. Dolečková, MD	Co-investigator	Charles University Prague
O. Klempířová, PhD	Co-investigator	Charles University Prague
O. Ulmanová, MD, PhD	Co-investigator	Charles University Prague
J. Roth, MD, PhD	Co-investigator	Charles University Prague
**Italy**		
F. Squitieri, MD, PhD	Principal investigator	IRCCS Casa Sollievo della Sofferenza Hospital
S. Maffi, MSc	Coordinating investigator	IRCCS Casa Sollievo della Sofferenza Hospital
E. Scaricamazza, MD, PhD	Co-investigator	IRCCS Casa Sollievo della Sofferenza Hospital
S. Migliore, PhD	Co-investigator	IRCCS Casa Sollievo della Sofferenza Hospital
M. Casella, MSc	Co-investigator	Italian League for Research on Huntington
**Ireland**		
J. Hoblyn, MD	Principal investigator	Bloomfield Hospital, Trinity College Dublin
M. Thangaramanujam, MISCP	Coordinating investigator	Bloomfield Hospital, Trinity College Dublin

*LUMC, Leiden University Medical Center; Topaz, Huntington Center Topaz Overduin; NeLL, National eHealth Living Lab; IRCCS, Istituto di Ricovero e Cura a Carattere Scientifico*.

* *Department of Public Health and Primary Care*.

$ *Department of Psychiatry*.

∇ *Department of Neurology*.

### Study Status

At time of submission of this manuscript, the study status is recruiting.

## Introduction

Huntington's Disease (HD) is a rare, autosomal dominant neurodegenerative disease characterized by progressive motor symptoms, cognitive impairments, and neuropsychiatric symptoms (1, 2). The disease is caused by a cytosine-adenine-guanine (CAG) repeat expansion in the *huntingtin* (HTT) gene (1). Reduced penetrance is seen in individuals with 36–39 CAG repeats, whereas individuals with >39 repeats will develop HD (1). HD affects an estimated 10.6–13.7 per 100,000 individuals in Western populations (3). Children with an HD affected parent have a 50% risk of inheriting the HD gene expansion. Clinical symptom onset is preceded by the premanifest stage (including the pre-symptomatic and the prodromal phase) (4, 5), in which subtle motor, cognitive and/or neuropsychiatric symptoms can already occur up to 10–15 years prior to the start of clear motor signs (4–6). After the onset of clinical motor changes (1, 6), which is still the “landmark” for manifest disease, life expectancy ranges between 15 and 20 years (2).

Disease onset usually occurs in the fourth or fifth decade of life (2, 6), when individuals are often very active in work, family and social life. As the disease progressively affects various functions essential for participation in everyday life activities, individuals become more dependent over time and the need for (long-term) care increases (1, 6). As no cure is available to date, current treatment strategies focus on symptom management and quality of life (QoL) maintenance (1, 3, 7). Previous studies have shown that HD greatly impacts the QoL of HD gene expansion carriers (HDGECs) (3, 8–10), even prior to symptom onset (3, 9). QoL tends to decline as the disease progresses over time (9, 11, 12), with individuals in the advanced stage often experiencing a worse QoL as compared to individuals at risk or in the premanifest stage (9, 11, 12). Partners of HDGECs also experience impaired QoL (13–15), especially with regard to coping, financial expenses, gaining access to care services, and perceived lack of knowledge from healthcare providers (HCPs) (13).

Due to the complex clinical nature of HD, there is an increasing need for comprehensive and multidisciplinary care services (16, 17), ranging from advice about genetic testing to palliative care. Although several HD expertise centers have been established across Europe (2, 18, 19), these specialist services often serve large geographical areas. Moreover, these services are not always instantly available, accessible or in close proximity to those seeking care (18). Other barriers impeding HD care provision include health care and transportation costs (2, 18). In addition, increasing physical limitations and burden, especially in the later stages of the disease, might pose difficulty in seeking and accessing care, leaving those with the greatest care need receiving the least care (20). To overcome these barriers, delivery of expert care should transcend geographical borders. More importantly, specialized professional care should be arranged and provided in such a way that HDGECs can live at home as long as possible while maintaining acceptable QoL. This results in the need for innovative ways to facilitate QoL maintenance in the home situation, primarily by increasing access to specialized professional care regardless of distance to care centers.

eHealth provides promising opportunities to facilitate such care, as it delivers and/or enhances health care services by using information and communication technologies (21). eHealth offers many possibilities, including home-based monitoring of health parameters, remote treatment options, as well as communication and information exchange between patients, family members and HCPs (20, 22). In addition, eHealth can increase care capacity by connecting experts to local clinicians remotely (20). Most importantly, eHealth allows for personalized, tailored care to be moved partially away from highly specialized centers (requiring patients to travel) into the patients' home (20, 22), which can be considered paramount for a disease as complex and rare as HD.

Although eHealth is considered promising in terms of acceptability, feasibility and effectiveness in other neurological and neurodegenerative diseases [e.g., dementia, Parkinson's disease (PD), multiple sclerosis] (23–30), research on eHealth (development) in HD is limited (31–35). A small-scale pilot study conducted in the Netherlands showed that an interactive knowledge website for HD patients and caregivers, combined with a videoconferencing tool (iQare), increased continuity of care as well as the quality of contacts between patients and informal/professional caregivers, without any travel time (31). eHealth was also found to be useful in conducting remote motor assessments and predictive testing services in HD (32–34), while maintaining quality of care and support (33).

In spite of the rapid growth in eHealth services more generally, (long-term) uptake of these services is often poor (36, 37) due to limited integration into the clinical workflow, reimbursement and legislation issues, and privacy and security issues (37–39). Limited uptake is also related to not actively involving end-users (e.g., patients) in an early stage of the design and development process (22, 37, 39). This may result in a lack of functionalities that are desired from a user perspective as well as in poor usability and user experience (37, 38, 40–42). Additional challenges in eHealth development reported in other neurodegenerative diseases, including PD, are the strong focus on motor aspects of the disease as opposed to important sources of disability reported by patients (e.g., depression, fatigue, and sleep disturbances), and the lack of user engagement (27, 29, 30).

A user-centered approach, in which end-users are closely involved in the design process, is therefore desired. It is assumed that this will lead to the necessary insight for developing eHealth that facilitates the patients' QoL, given its suitability as shown in other neurological and neurodegenerative diseases (43–47). Actively involving both HDGECs and their partners, and addressing their needs is important especially in HD given the complexity and diversity of symptoms and the variety in needs experienced in different stages of the disease, including pre-symptomatic and prodromal stages. In addition, partners of HDGECs often have the tendency to neglect their own experiences and needs (15), yet these should not be overlooked. As a high level of unmet needs for health and social services can negatively impact health-related QoL in HD (12), it is important to include the needs of both HDGECs and their partners in the development of eHealth technologies. By also actively involving HCPs with expertise in HD, disease-specific characteristics, in particular neuropsychiatric and cognitive impairments (18), can be considered in advance in the eHealth development process.

To date, no HD-specific needs assesment exists in relation to QoL from the perspective of HDGECs and their partners. To ensure high-quality remote care services that increase the QoL of HDGECs and their partners across Europe, it is therefore paramount to include the perspective of HDGECs, their partners and HCPs in designing and developing an eHealth platform. A participatory user-centered design (UCD) approach ensures the inclusion of these perspectives by closely involving end-users in the development process, thereby increasing the probability of a good fit between the eHealth platform and end-users' needs, wishes, and daily activities (39, 48, 49). In the present study we will use the principles of UCD to develop an innovative eHealth platform to facilitate QoL in HDGECs and partners across Europe. This may be digital information solutions (e.g., websites, apps, online videos), digital communication tools (e.g., sensors, questionnaires, chat messaging), and/or digital support tools (e.g., shared agendas, notification systems). In this article we outline a detailed description of the aim, design and study procedures of the HD-eHelp study.

### Study Aim and Objectives

The HD-eHelp study aims to capture the needs and wishes of HDGECs, partners and HCPs in order to develop a European eHealth platform following the principles of UCD (48). The specific study objectives are, to:

1. explore and map desires, needs and experiences of HDGECs, partners, and HCPs in relation to QoL, HD care and eHealth;
2. identify eHealth opportunities and strategies to fulfill these desires and needs;
3. identify design requirements for an eHealth platform in collaboration with end-users to ensure desirability, and;
4. develop prototypes of the eHealth platform with end-users to create a positive user-experience.

## Methods and Analysis

### Study Design

We will employ a UCD approach (48) in which an understanding of the end-users' needs, preferences and contexts is pivotal. In our study, we will include HDGECs, partners and HCPs as end-users. To optimally align the eHealth platform with their needs and wishes, end-users will participate in all three phases of the research and development process (i.e., 1. Exploration; 2. Concept development; 3. Prototype testing). As user-centered (participatory) design approaches have not been extensively used or described in HD (34, 50), we provide a detailed description of how we adjusted this approach to the multiple target groups and multinational nature of our study in the procedure section.

This study will be coordinated from the Netherlands. Research sites from the Netherlands, Germany, Czech Republic, Italy, and Ireland (see Table 1) will be involved to develop and test a multilingual platform that is suitable within different healthcare systems and cultural contexts. The duration of the study will be ~18 months, including preparation, data analysis and prototype development. A large-scale evaluation of the platform in a randomized controlled trial (RCT) is scheduled, yet beyond the scope of this study protocol.

### Study Sample and Recruitment

Premanifest (preHD) and manifest (mHD) gene expansion carriers who are living at home, partners of HDGECs, and HCPs will be invited to participate in this study. Table 2 provides an overview of the eligibility criteria for each participant group.

**Table 2:** Eligibility criteria for study participants.

	**Inclusion criteria**	**Exclusion criteria**
HDGECs	• Genetically confirmed HD (i.e., CAG ≥ 36) • No clinical motor features (i.e., UHDRS DCL < 4) in case of premanifest HDGECs. Clinical motor features (i.e., UHDRS DCL = 4) in case of manifest HDGECs • Age ≥ 18 years • Living at home • Proficient in language of respective country • Current participation in Enroll-HD • Ability to attend study sessions	• Having a partner that participates in this study • Being a FPEP member of this study • Any present serious psychiatric, neurological, sensory, or any other comorbid disorders known to influence participants' judgements and therefore likely to affect the needs and desires experienced as well as the ability to assess eHealth use (as judged by clinical team) • Inability to give consent
Partners	• Spouse or partner of premanifest or manifest HDGECs • Living together with premanifest or manifest HDGECs • Age ≥ 18 years • Proficient in language of respective country • Ability to attend study sessions	• Being an HDGECs themselves (as confirmed by genetic test) • Having a partner that participates in this study • Being a FPEP member of this study • Any present serious psychiatric, neurological, sensory, or any other comorbid disorders known to influence participants' judgements and therefore likely to affect the needs and desires experienced as well as the ability to assess eHealth use (as judged by clinical team) • Inability to give consent
Health care providers	• Providing HD care ≥ 2 years • Age ≥ 18 years • Proficient in language of respective country • Ability to attend study sessions	• Inability to give consent

*HDGECs, Huntington's Disease gene expansion carriers; HD, Huntington's Disease; CAG, cytosine-adenine-guanine repeat length; UHDRS, Unified Huntington's Disease Rating Scale; DCL, Diagnostic Confidence Level; FPEP, Family Patient Expert Panel*.

We aim to recruit HDGECs from the locally held Enroll-HD database (https://www.enroll-hd.org/) at the respective research sites (51). Enroll-HD is a global, on-going observational study for families affected by HD, collecting longitudinal data on disease characteristics and progression (51). With over 20,000 participants enrolled worldwide, Enroll-HD provides a large database of HDGECs for which phenotype and genotype are well-established, providing additional context for mapping their desires and needs across different disease stages in this study. HDGECs are not required to have a spouse/partner to participate in this study and will be selected to cover a wide range of disease stages.

Partners (i.e., spouses or unregistered/unmarried partners of premanifest and manifest HDGECs) and HCPs will be recruited from clinics, HD centers, patient groups and via the research sites' primary and secondary care networks in the respective countries. The HCP sample will represent professionals who work in HD expertise centers and will be selected to cover all major expertise that is involved in HD care (e.g., neurologists, psychiatrists, psychologists, social workers, speech and swallowing therapists, dieticians, occupational therapists, and physiotherapists).

Potentially eligible participants will be informed about this study (face-to-face or by telephone) by the coordinating researcher(s) at each site and will receive a study information package, if interested. After a minimum period of 7 days, HDGECs will be contacted again and provided with additional information or clarification, if needed. Partners and HCPs can indicate their interest in participation via a response card. Participants will be invited to the first study activity upon receival of the signed informed consent forms. All participants will receive travel reimbursement, if applicable.

### Sample Size

We estimated the number of participants based on guidelines for user testing (52), thereby correcting for anticipated attrition rates. Five participants generally find 80 percent of all problems when testing a moderately complex concept or prototype. This is more than enough for developing an eHealth platform that can be tested in an RCT. However, as the target users (i.e., patients and partners) of the intended eHealth platform are experiencing very diverse stages of the disease, we include at least 7 participants per stage (i.e., preHD or mHD) per target group. The study design allows participants to take part in multiple phases. If they are unwilling or unable to participate in subsequent phases, additional participants will be recruited until data saturation (i.e., no new insights emerging from newly collected data) is reached within each study phase (53).

Table 3 provides the estimated number of participants per phase and per country throughout the study. In total, we aim to include 20 HDGECs (10 preHD; 10 mHD), 20 partners (10 of preHD; 10 of mHD) and 20 HCPs in the Netherlands. In Germany, Czech Republic, Italy, and Ireland we aim to include 14 HDGECs (7 preHD; 7 mHD), 14 partners (7 of preHD; 7 of mHD) and 14 HCPs per country. This will result in the inclusion of 76 HDGECs (38 preHD; 38 mHD), 76 partners (38 of preHD; 38 of mHD) and 76 HCPs throughout the whole study.

**Table 3:** Estimated number of participants per UCD phase per country.

	**NL**	**GE**	**CZ**	**IT**	**IE**	**Total**
**PHASE 1: EXPLORATION**						
**Interviews**						
HDGECs						**36**
preHD	6	3	3	3	3	
mHD	6	3	3	3	3	
Partners						**36**
preHD	6	3	3	3	3	
mHD	6	3	3	3	3	
**Focus groups**						
HCPs	12	6	6	6	6	**36**
**Total phase 1**	**36**	**18**	**18**	**18**	**18**	**108**
**PHASE 2: CONCEPT DEVELOPMENT**						
**Co-creation sessions**	^*^	–	–	–	–	^*^
**Concept testing**	^*^	–	–	–	–	^*^
**Total phase 2**	^*^	**–**	**–**	**–**	**–**	^*^
**PHASE 3: PROTOTYPE TESTING**						
**Prototype testing**	^*^	–	–	–	–	^*^
**“Think-aloud” sessions**						
HDGECs						**40**
preHD	4	4	4	4	4	
mHD	4	4	4	4	4	
Partners						**40**
preHD	4	4	4	4	4	
mHD	4	4	4	4	4	
HCPs	8	8	8	8	8	**40**
**Total phase 3**	**60**	**24**	**24**	**24**	**24**	**156**
**Study total**	**60**	**42**	**42**	**42**	**42**	**228**

*UCD, User-centered design; NL, the Netherlands; CZ, Czech Republic; GE, Germany; IT, Italy; IE, Ireland; HDGECs, Huntington's Disease gene expansion carriers; preHD, premanifest HDGECs; mHD, manifest HDGECs; HCPs, health care providers*.

* *In the Netherlands, the same individuals that participated in the exploration phase (phase 1) will be asked to participate during co-creation and concept testing (phase 2), and prototype testing (phase 3). The bold values were meant to highlight the total sample size (as the bold values are the sum of the sample sizes of each individual study group)*.

### Study Procedures

The different study procedures for each UCD phase, and how we adjusted these to the multinational nature and target groups of our study, are described in detail below. As the study is coordinated from the Netherlands, Dutch end-users will be involved in all phases of the development process. Due to feasibility and time constraints, end-users in the other participating countries will only be involved in phase 1 and 3 (see Table 3). This is considered sufficient to design and adapt the eHealth platform to each language and healthcare system. To prevent a loss of lingual and cultural aspects in concept development during phase 2, the international project team and an international Family Patient Expert Panel (FPEP) will be actively involved throughout phase 2. The panel consists of one representative from each participating country, appointed by the respective national HD association. In addition, two people from the patient advocacy group in the European Reference Network for Rare Neurological Diseases participate to have additional disease groups represented.

Throughout the study, regular meetings with the international project team, a Dutch advisory board of HCPs and the FPEP will provide guidance to the research and design process. The HCP advisory board and the FPEP will review study procedures and materials to ensure suitability and comprehensibility. Prior to the start of the study in each country, all relevant study materials will be translated into the respective languages. Guidelines for all study sessions have been developed. Study sessions will be audio recorded and conducted by trained staff.

#### Phase 1: Exploration

We will gather an in-depth understanding of end-users' needs, desires and experiences regarding QoL, HD care, and eHealth possibilities using interviews and generative techniques. As compared to more conventional qualitative techniques (i.e., interviews and focus groups), generative techniques (54), such as sensitizing assignments and journey mapping, can help to facilitate a deeper level of understanding (55) and access people's tacit knowledge (i.e., easy to act upon but difficult to express in words) and latent knowledge (i.e., not yet aware of) (56). As desires, needs and experiences are often concealed in these deeper levels of knowledge, these techniques provide access to the user's hidden world (55) and at the same time help to build empathy during the design process (57).

**Figure 1:**
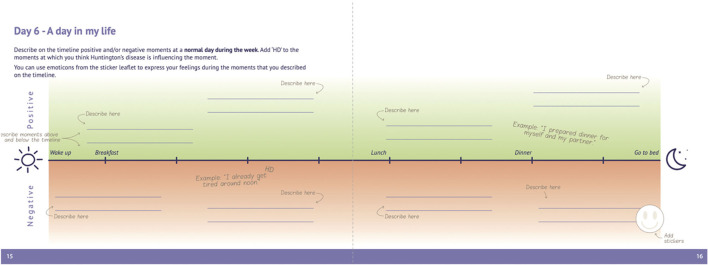
Image of assignment in sensitizing booklet for manifest HDGECs.

All participants will complete a workbook consisting of sensitizing assignments that encourage them to reflect on their routines, habits and feelings regarding HD and QoL (e.g., current/future complaints, important conversations and locations, housing situation and tools used, reflection of a day in their lives). This awareness helps to express their experiences and needs during semi-structured interviews (in case of HDGECs and partners) and focus groups (in case of HCPs). Participants will be asked to complete an assignment every day (~10 min per day) for a total of 7 days at home (HDGECs/partners) or at work (HCPs). Participants can receive daily reminders by phone/e-mail upon request. The sensitizing assignments have been co-developed with the HCP advisory board/FPEP and have been adapted and tailored to each participant group. To ensure suitability and comprehensibility, the assignments for HDGECs were pilot tested with one premanifest and one manifest HDGEC (and spouse). This resulted in some important adjustments for the workbooks for manifest HDGECs, including for example landscape instead of portrait orientation, larger font size, more writing space, and less suggestive examples to avoid copying (see Figure 1 for an example of an assignment in the workbook for manifest HDGECs). Together with the researchers from the respective sites, the final workbooks were adapted to each language.

The interviews with HDGECs and partners are aimed at understanding participants' daily experiences with HD (caregiving) and their perceptions of QoL. Additionally, participants will be asked to voice their hopes and dreams for the future regarding QoL. Interviews will take ~1.5 h. As opposed to the face-to-face sessions often seen in UCD, the interviews and focus groups will be mainly conducted through online videoconferencing due to the restrictions raised by the current COVID-19 pandemic. In the case of connectivity issues, participants will be interviewed by telephone. HCPs will be asked to talk about their daily work experiences with HD during focus group sessions of ~2 h (including breaks). Generative techniques such as patient journey mapping (58) will be applied to gain in-depth insights into the experiences of HCPs regarding HD treatment and challenges and opportunities in providing HD care. Focus group sessions will consist of a maximum of six HCPs per session and will be preferably conducted online. The groups will consist of HCPs with varying expertise in the respective countries.

HD experts from the participating research sites will provide additional information on available HD care services and the current use of eHealth technologies in HD care in their respective country to complement the information given by participants.

#### Phase 2: Concept Development

Based on the data gathered in phase 1 from all participating countries, we will develop concepts (through descriptions and visualizations) of the eHealth platform together with the same Dutch participants that participated in the first phase via sensitizing assignments and co-creation sessions. The sensitizing assignments will follow the same procedures as described previously. The focus in this phase will be on eHealth opportunities and possibilities (e.g., an app providing tailored practical information or a website facilitating a buddy system), yet the exact content depends on the output gathered during phase 1. The assignments will be co-developed with the HCP advisory board/FPEP and will be pilot tested with HDGECs.

**Figure 2:**
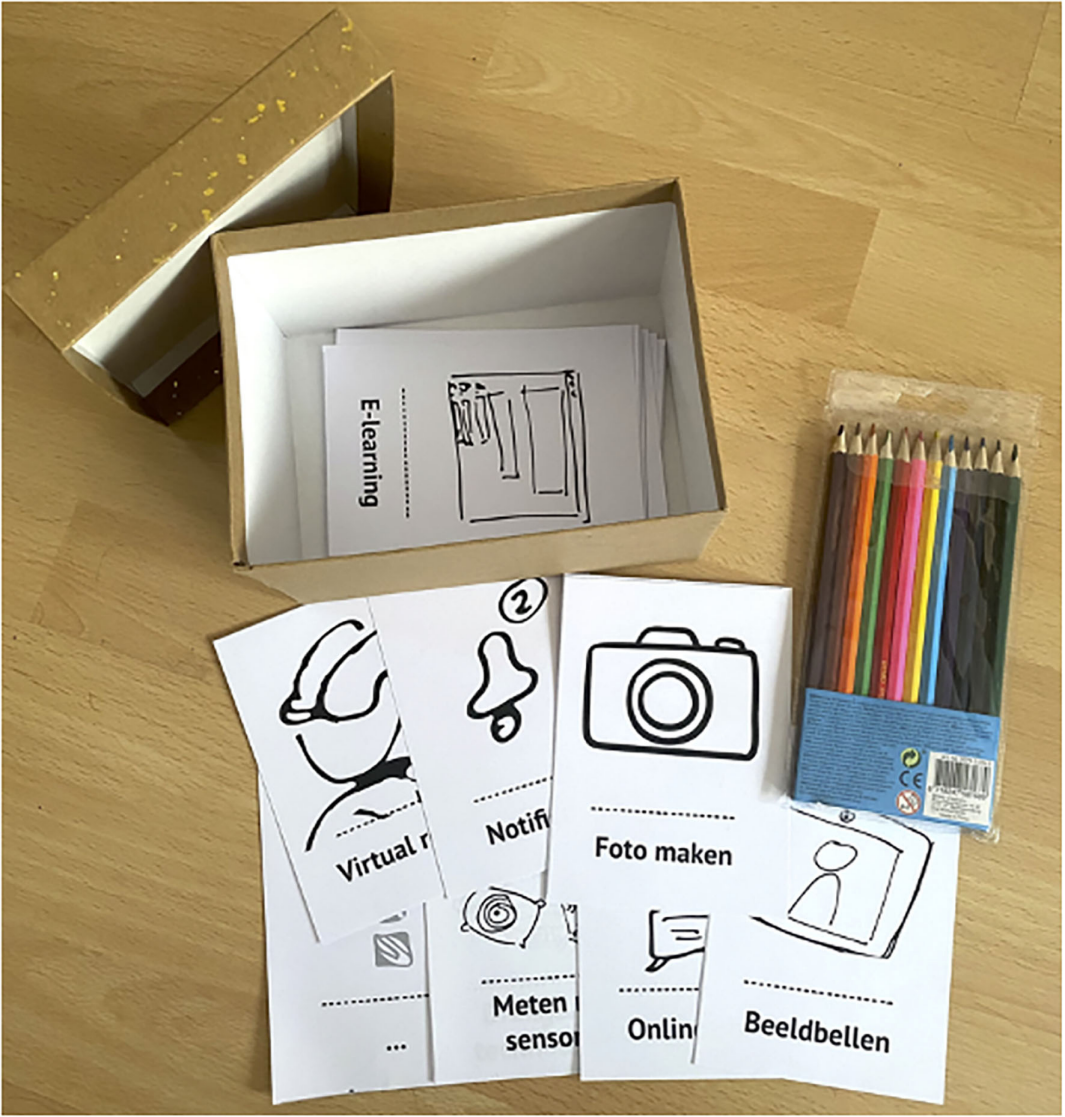
Example of a tailor-made toolkit.

During the co-creation sessions with each end-user group, problems and opportunities for eHealth, as well as solutions to address these, will be identified for all groups. Generative techniques (e.g., making a collage, journey mapping, and/or mind mapping) will be used to gather information on needs, motivations, and wishes that might not be easily expressed in words. Participants will work with tailor-made toolkits (54) consisting of, for example, paper templates, physical objects, and stickers with words and images that participants can use to reflect on their experiences with HD, generate ideas for solutions, and share them with each other (see Figure 2 for an example of a toolkit used during a research presentation at a Huntington café). The toolkit materials will be developed and pilot tested to match the participants' cognitive and motor skills. For example, too much material on a table could cognitively overload participants who have problems with executive functioning. The co-creation sessions will generally consist of six participants and may take ~3 h (including breaks). The sessions are intended to be performed physically, yet could also be performed online depending on the COVID-measures at the time.

The outcomes will be further developed by the research team into several detailed concepts of the eHealth platform, such as a regular monitoring service or notifications on the mobile phone. These concepts will be evaluated by the same participants during individual concept evaluation sessions of ~1 h either at the respective site or online. Low fidelity mock-ups representing concept features will be used to evaluate the user interaction in practice. For example, the use of a mobile application will be mimicked by a paper representation of the interface in which interface elements are manually altered. The concepts will be evaluated on desirability, user-experience, and expected effect on QoL via questions and short interviews. Participants will be asked to state their preferences regarding the concepts and evaluate the concepts' fit into their daily lives. A researcher will observe the use and interaction with the concept and take notes of the interaction. The evaluation will result in a redesign of the concept and design recommendations. During this process, the international project team and the FPEP will be actively involved to evaluate concepts on their fit in the different cultures and health systems. This phase can be considered as an iterative process of designing, evaluating, selecting and adjusting concepts (59), which ends with definitive design choices for prototypes of the eHealth platform (e.g., a definitive decision on functionalities and scenarios how the platform is envisioned to be used).

#### Phase 3: Prototype Testing

High-fidelity prototypes (i.e., digital representations of a product with close resemblance to the final design), as developed by the research team, will be reviewed by the same end-users that participated in the previous phase to assess usability and user-experience. The procedures for prototype testing are similar to those described above for concept evaluation. The participants' responses will be used to remove all major usability issues, such as an unclear navigation structure, and refine the user experience (e.g., a friendly or professional look and feel) in an iterative process. The same participants may be invited to perform a second round of evaluations on the improved prototype. Each individual prototype evaluation session will be held either at the respective site or online, and will take 1 h (with a minimum of 2 weeks in between, if applicable).

As soon as the major usability issues of a prototype are solved and the participants approve its usefulness, a pre-final prototype will be translated into the respective language. Newly recruited participants in each country will then evaluate the prototype during individual “think-aloud” sessions. Each session will take ~90 min and will be preferably held at home (HDGECs/partners) or at work (HCPs). End-users will be asked to use and explore the prototype, reflect on their experience, and express their thoughts (e.g., “What's this button for?” or “Strange picture”) in the presence of a researcher. We will explore perceived usefulness, perceived ease of use, and intention to use. The findings from testing the pre-final prototype will result in a design proposal, which will then be further developed into a fully functional eHealth platform that can be tested in an RCT study.

### Additional Measures

To describe the population under study and to provide additional context to the insights gained, we will collect sociodemographic information (e.g., gender, age, work situation) from all participant groups via self-report questionnaires provided with the sensitizing assignments. In addition, care-related information will be collected from partners (e.g., years of taking care of HD affected partner, care tasks) and HCPs (e.g., profession, years of working with HD).

For HDGECs we aim to also collect sociodemographic, clinical, neuropsychiatric, and cognitive data from their last or upcoming Enroll-HD visit. More particularly, we will collect, amongst others, CAG repeat length, clinical motor features of HD [based on the Unified Huntington's Disease Rating Scale (UHDRS) Total Motor Score], years of HD diagnosis (as reported by rater), independence and functioning in daily living (UHDRS Total Functional Capacity), functional status (UHDRS Independence Scale), current medication use, behavioral problems (Problem Behaviors Assessment), symptoms of anxiety and depression (Hospital Anxiety and Depression Scale), presence of suicidal ideation/behavior (Columbia Suicide Severity Rating Scale), health-related quality of life (Short Form Health Survey 12), cognitive state (comprehensive neuropsychological battery consisting of, amongst others, Symbol Digit Modalities Test, categorical verbal fluency and the Stroop Test). This additional information will provide a better understanding of specific disease characteristics and symptoms for mapping the desires and needs across different disease stages. Detailed information on the Enroll-HD measures and (informed consent) procedures are described elsewhere (51).

### Data Analysis

The collected sociodemographic and clinical data will be used to describe the population under study and to provide additional context to the insights gained in each phase. A brief description of the planned data analysis corresponding with the specific procedures per phase is provided below.

#### Phase 1: Exploration

Qualitative data, consisting of transcripts of interviews/focus groups, field notes and other materials (e.g., filled out sensitizing assignments) will be analyzed and interpreted using thematic and on the wall analysis (54). During on the wall analysis, the office wall will be used as a large spreadsheet to label and categorize all data into clusters of themes and insights. In addition to thematic analysis, this allows the researchers to absorb the richness of the data in an unconstrained manner, which is beneficial for exploring the opportunities of eHealth through a non-linear thinking process (60). To make sure that all basic elements of QoL are covered in our findings, we use the six dimensions of positive health (61) as a benchmark: bodily functions, mental functions and perceptions, spiritual dimension, quality of life, social and societal participation, and daily functioning.

Findings from these analyses on the needs, desires, and experiences of end-users regarding QoL and HD care from all countries will be visualized in a patient journey map by the research team (58). This is a detailed schematic representation of phases and events in relation to HD and the people involved. This will be presented to the HCP advisory board, the FPEP and the international project team to align with (clinical) expert and end-user perspectives.

#### Phase 2: Concept Development

The HD patient journey and eHealth ideas resulting from all countries during phase 1 will be used to identify possible problem areas and points of innovation that may improve the end-user's QoL. Depending on the findings of phase 1, this may for example be to support care provision already before the onset of symptoms. Through generative sessions by the research team and the previously described co-creation sessions, ideas will be generated to address the identified problem areas and points of innovation. The resulting ideas will be clustered into a list of eHealth opportunities and strategies. Subsequently, eHealth concepts will be developed together with end-users in the co-creation sessions. Based on the qualitative data of the co-creation sessions, including transcripts, field notes and other study materials (e.g., output of sessions), low fidelity mock-ups representing concept features will be developed and evaluated. Qualitative data arising from the concept evaluation sessions will be reviewed and clustered in themes by the research team to define the design requirements for an HD eHealth platform prototype. The international project team and the FPEP will be closely involved to provide feedback and evaluate concepts on their fit in the different cultures and languages.

#### Phase 3: Prototype Testing

The transcripts and field notes resulting from the prototype testing sessions will be reviewed and clustered in themes by the research team to remove major usability issues and refine the user experience. Qualitative data of the “think-aloud” sessions will be clustered on problem severity in order to provide a comprehensive overview of the strengths and weaknesses of the HD eHealth platform prototype. This will be used to identify critical problems to be addressed in the development of the final prototype.

Data from all countries will additionally be analyzed to evaluate the suitability of the HD eHealth platform within different healthcare systems and acceptability within different cultural contexts. Any additional information or necessary changes resulting from this analysis will be incorporated.

### Data Handling and Storage

Data will be handled confidentially. All study data will be processed, stored and disposed of in accordance with the General Data Protection Regulation (62) and all applicable legal and regulatory requirements at the respective sites. Study sessions will be audio recorded with permission of the participants. Audio recordings will be stored on a secure disk at the respective site and will be deleted as soon as the design process is finished. Each study session will be transcribed using intelligent verbatim transcription. Non-English transcripts and study materials will be translated to English by researchers at the respective sites.

Data collection will be performed within a secured electronic case report form (eCRF). To allow secure data management and transmission between countries, the eCRF will be implemented in a Data Management System accessible at the respective sites in each country. The database will only be accessible for authorized personnel via a unique login and user ID. All participant data will be pseudonymized and identifiable data (e.g., name, e-mail, address) will be removed prior to uploading. All source documents will be stored in a secure closet for a certain period of time depending on the legal and regulatory requirements at each site.

### Benefits and Risks Assessment

No risks or ethical concerns are anticipated. (Serious) adverse events are not expected due to the non-invasive character of the study. Participants will reflect on their own needs, desires and expectations regarding the disease and eHealth features during this study. This might potentially cause distress as it may be of sensitive nature for some participants given the vulnerability of this group. The researchers will be experienced and will offer the participant the opportunity to talk to an HCP in case the participant will become distressed. At the same time, these potentially unfavorable effects will be minimized by pilot testing all materials and study sessions, by mainly focusing on personal benefits and accomplishments in their management of HD and by engaging them in the development of the eHealth platform.

## Discussion

As a result of the neurodegenerative nature of HD, the disease causes a progressive decline in functioning and significantly influences the QoL of both HDGECs and their partners (3, 8–10, 13). Despite the emergence of HD expertise centers across Europe (2, 18, 19), we continue to face some important barriers to HD care provision, including additional costs, geographic distance of centers and availability/accessibility of these services in general (2, 13, 18). As the disease progressively advances over time, challenges to seeking or accessing care might arise (e.g., physical limitations, increased burden, cognitive/neuropsychiatric impairments), leaving those with the greatest care need behind (20). eHealth provides promising opportunities to overcome these barriers by improving the accessibility of care. In the present study, we initiated an innovative UCD study to capture the needs and wishes of HDGECs, partners and HCPs in order to develop an eHealth platform targeting QoL. The eHealth platform will be co-developed with these end-users by actively involving them throughout each stage of the design process, thereby tailoring remote HD service provision to the unique requirements of HDGECs, partners and HCPs. Given the rare nature of HD, we aim for an innovative European platform which allows for remote treatment options, information exchange and connection of experts to local clinicians beyond regional and national borders.

Some challenges might arise during the design process, including differences in healthcare systems, HD care provision and cultural context in all countries involved in this study. We will address these differences by actively involving the international project team, the FPEP, and end-users from each participating country in all design decisions. Furthermore, differences in clinical presentations and needs at different stages of the disease might pose additional challenges in developing a European platform that is both applicable and generalizable to all participants. By including HDGECs and partners across different disease stages, as well as HCPs with expertise in HD, we will be able to gather an overall understanding of the needs and desires experienced by these groups. The flexibility of UCD allows us to address a variety in needs in different ways, for instance, by 1. designing universally, 2. designing more modularly so that specific features can be added when needed, or 3. designing for specific target groups within the study population. As the HD community is very motivated and willing to participate in research, we do not expect challenges with accrual of sufficient participants in each country. Lastly, online study sessions as opposed to face-to-face sessions might pose some challenges, including connection issues, difficulties in capturing non-verbal communication, or perceived (emotional) distance between participant and researcher. We pilot-tested the sensitizing assignments and online interviews with a premanifest and manifest HDGECs (and spouse) and both agreed that online interviews were convenient and saved travel time. None had difficulties with setting up the connection, yet technical issues should be considered and an alternative way to conduct the interview (such as calling by phone) should be present prior to starting the interviews. At the same time, conducting these sessions remotely already provides a great opportunity for participants to experience online services, which greatly fits with the nature of what we are designing. In addition, this could be beneficial later on when using telehealth services, especially in the light of the shift toward a more blended care approach due to the ongoing COVID-19 pandemic (63).

Some unique aspects of this study are worth mentioning as well. Although eHealth seems promising in other neurological and neurodegenerative diseases (e.g., dementia, Parkinson's disease, multiple sclerosis) (23–30), the studies examining the benefits of eHealth in HD are limited (31–34) and often hampered by methodological challenges. In particular, these studies failed to use telehealth systems specifically designed to fit the unique perspectives and needs of HDGECs and their families, which can ultimately affect uptake later on (22, 37, 39). Actively involving end-users and addressing their needs when designing eHealth applications can be considered crucial in a disease as complex and rare as HD. In UCD, the end-user's needs are key in the choice and design of features of an application, which is important given the devastating challenges these people face and the variety in needs they might experience. Another unique aspect of this study is the inclusion of a partner group. HD does not only affect the individual but also the people in their environment. Partners of HDGECs have their own experiences and needs with regard to their QoL that should not be overlooked. Moreover, as HD already has a tremendous impact on the QoL and needs/wishes of those affected in an early stage (e.g., tested gene positive, no symptoms) (3, 9, 11), we believe it is paramount to include the perspectives and needs of both HDGECs and their partners across different disease stages in this innovate UCD approach. By also actively involving HCPs with expertise in HD, some additional challenges that might arise in engaging HDGECs in eHealth applications can be considered in advance, such as progression in symptoms that impact needs and wishes experienced, apathy, anosognosia, denial (18), or motor impairments. Including HCPs also ensures that relevant clinical expertise will be integrated, and, at the same time, stimulates the future dissemination of knowledge and connection of experts. The eHealth platform will therefore be tailored to the unique requirements of HDGECs, partners and HCPs. We expect that this will greatly benefit future uptake, as functionalities that are desired from a user perspective will be included, and user experience and usability of the platform will be tested (37, 38, 40–42).

To conclude, an innovative European eHealth platform for HDGECs and partners will be developed based on their needs, wishes and desires, following a UCD approach. This approach allows for a personalized, tailored care platform suitable to all languages and different healthcare systems involved. After development of the platform, the eHealth intervention will be evaluated on effectiveness, feasibility and user experience in an RCT (this is not part of the UCD protocol as described in this article). We expect that an eHealth platform will enhance current HD supportive care services across Europe by making high-quality care accessible outside specialized centers (requiring individuals to travel) in the participants' homes, thereby circumventing still existing barriers in HD care provision. We intend to implement the platform, provided that the evaluation shows positive results, to ensure free availability for patients and their partners after the study. As HD is a very complex and rare disease, future studies in other rare diseases might also benefit from the adaptations to, and the results of, the participatory UCD approach described here when designing and developing eHealth applications to enhance their supportive care services worldwide.

## Ethics Statement

The study was cleared for Ethics by the medical research Ethics committee of Leiden Den Haag Delft in the Netherlands (file number: N20.013). For the other research sites, Ethical approval was obtained by the local Ethics committees in the respective countries (i.e., Germany, Italy, Czech Republic, and Ireland). This study will be conducted in accordance with the principles of the Declaration of Helsinki (64) and the General Data Protection Regulation (62). Written informed consent will be obtained from all participants prior to any study-related activities.

## Author Contributions

EM and NC were involved in initiating and conceptualizing the project together with the HEALTHE-RND consortium, as well as funding acquisition. EM, NV, A-WH, and PL drafted the protocol. EM, NV, A-WH, PL, ED, SB, and NC were major contributors in drafting and finalizing the study protocol. PL drafted the manuscript. EM, NV, A-WH, ED, SB, NC, and WF contributed to the content and revised sections of the manuscript. All authors contributed to the article and approved the submitted version.

## Funding

This study was funded by the EU Joint Programme Neurodegenerative Disease Research (JPND) (grant number: 01ED1903). The Dutch local grant provider is the Netherlands Organisation for Health Research and Development (ZonMw) (project number: 733051085). The funders had no role in study design, data collection and analysis, interpretation of data, decision to publish, or preparation of the manuscript.

## Collaborating Author Names Healthe-RND Consortium

### The Netherlands

Niels H. Chavannes, Eline Meijer, Anne-Wil Heemskerk, Erik van Duijn, Susanne T. de Bot, Niko J. H. Vegt, Pearl J. C. van Lonkhuizen, Stephanie Feleus, Esther C. Arendts, Amy Putman.

### Germany

G. Bernhard Landwehrmeyer, Alzbeta Mühlbäck, Wiebke Frank, Katrin S. Lindenberg, Nana Kovacevic, Rainer Hoffmann, Michael Bachmaier, Peter Brieger.

### Czech Republic

Jiří Klempíř, Romana Konvalinková, Eva Bezuchová, Kristýna Dolečková, Olga Klempířová, Jan Roth, Olga Ulmanová.

### Italy

Ferdinando Squitieri, Sabrina Maffi, Eugenia Scaricamazza, Melissa Casella, Simone Migliore, Marta Tommolini, Barbara D'Alessio.

### Ireland

Jennifer Hoblyn, Muthukumaran Thangaramanujam.

### Scotland

Peter Foley, Lewis Killin, Jacqueline Kerr.

### United Kingdom

Stephen McKenna, Ian McKenna, Jeanette Thorpe, Alice Heaney, Anna Coffey, Rhona MacLeod, Ramona Moldovan.

## Conflict of Interest

The authors declare that the research was conducted in the absence of any commercial or financial relationships that could be construed as a potential conflict of interest.

## Publisher's Note

All claims expressed in this article are solely those of the authors and do not necessarily represent those of their affiliated organizations, or those of the publisher, the editors and the reviewers. Any product that may be evaluated in this article, or claim that may be made by its manufacturer, is not guaranteed or endorsed by the publisher.

## Abbreviations

HD: Huntington's disease
HTT: *huntingtin* gene
CAG: cytosine-adenine-guanine repeat length
QoL: quality of life
HDGECs: Huntington's Disease gene expansion carriers
HCP: health care providers
PD: Parkinson's Disease
UCD: user-centered design
preHD: premanifest HDGECs
mHD: manifest HDGECs
FPEP: Family Patient Expert Panel
UHDRS: Unified Huntington's Disease Rating Scale
eCRF: electronic case report form.
